# The future of CRISPR in *Mycobacterium tuberculosis* infection

**DOI:** 10.1186/s12929-023-00932-4

**Published:** 2023-05-27

**Authors:** Rima Zein-Eddine, Guislaine Refrégier, Jorge Cervantes, Noemí Kaoru Yokobori

**Affiliations:** 1 Laboratoire d’Optique et Biosciences (LOB), Ecole Polytechnique, Route de Saclay 91120, Palaiseau, France; 2 Université Paris-Saclay, CNRS, AgroParisTech, Ecologie Systématique et Evolution, 91190 Gif-Sur-Yvette, France; 3 Paul L. Foster School of Medicine, Texas Tech University Health Sciences Center, El Paso, TX 79905 USA; 4 Servicio de Micobacterias, Instituto Nacional de Enfermedades Infecciosas (INEI)-ANLIS and CONICET, C1282AFF Buenos Aires, Argentina; 5 Consejo Nacional de Investigaciones Científicas y Técnicas (CONICET), Buenos Aires, Argentina

**Keywords:** CRISPR-Cas, *Mycobacterium tuberculosis*, MTBC, Spoligotyping, CRISPRi, Functional genomics, Diagnostic methods

## Abstract

Clustered Regularly Interspaced Short Palindromic repeats (CRISPR)-Cas systems rapidly raised from a bacterial genetic curiosity to the most popular tool for genetic modifications which revolutionized the study of microbial physiology. Due to the highly conserved nature of the CRISPR locus in *Mycobacterium tuberculosis*, the etiological agent of one of the deadliest infectious diseases globally, initially, little attention was paid to its CRISPR locus, other than as a phylogenetic marker. Recent research shows that *M. tuberculosis* has a partially functional Type III CRISPR, which provides a defense mechanism against foreign genetic elements mediated by the ancillary RNAse Csm6. With the advent of CRISPR-Cas based gene edition technologies, our possibilities to explore the biology of *M. tuberculosis* and its interaction with the host immune system are boosted. CRISPR-based diagnostic methods can lower the detection threshold to femtomolar levels, which could contribute to the diagnosis of the still elusive paucibacillary and extrapulmonary tuberculosis cases. In addition, one-pot and point-of-care tests are under development, and future challenges are discussed. We present in this literature review the potential and actual impact of CRISPR-Cas research on human tuberculosis understanding and management. Altogether, the CRISPR-revolution will revitalize the fight against tuberculosis with more research and technological developments.

## Introduction

Clustered Regularly Interspaced Short Palindromic repeats (CRISPR) were first identified in 1987 by Ishino and coworkers in *Escherichia coli* as unique loci with the repeated occurrence of so-called “direct repeats” [1–3]. Similar sequences were identified in 1993 by Groenen et al. in *Mycobacterium tuberculosis* and by Doran et al. in *M. bovis* [4, 5]. This discovery promoted the setup of spoligotyping, a method for exploring *M. tuberculosis* complex (MTBC) diversity [6]. It was not until the mid to late-2000s that the function of these enigmatic repeats present in most prokaryotes was described as their adaptive immunity system [1]. However, little attention was paid at that time to the CRISPR locus in *M. tuberculosis*, other than as a phylogenetic marker. With the discovery of **C**RISPR-**as**sociated proteins (the Cas proteins) and their role in the recognition and processing of nucleotide sequences, from a mere bacterial genetic curiosity, CRISPR-Cas systems rapidly raised to the most popular tool for genetic modifications which revolutionized the study of microbial physiology [7–9]. *M. tuberculosis* was not an exception this time and related research is slowly growing. Tuberculosis still affects millions of patients worldwide despite the continuous scientific and technological advances [10] and CRISPR-based technologies are expected to boost our understanding and improve the management of this ancient disease.

We here present a literature review of the potential and actual impact of CRISPR-Cas research in the tuberculosis field. To build a whole picture, we will thus first present the structure of internal CRISPR-Cas and its possible roles on MTBC physiology. We will then discuss the technological developments based on CRISPR-Cas, for diagnostics of tuberculosis and for targeted genetic modifications in research.

## CRISPR-Cas: the ABC

CRISPR systems play a role in prokaryote immunity through two main components: the CRISPR locus which is transcribed and then cleaved to generate short CRISPR RNAs (crRNAs), and the Cas proteins encoded alongside. Cas proteins are a family of nucleases that cleave nucleotide sequences at specific sites [11]. When a virus infects the cell, the crRNAs guide the Cas proteins to cleave RNA or DNA sequences that are complementary to the spacer acquired in a previous encounter with the invader [12, 13]. Self-immunity is prevented through the recognition of the protospacer adjacent motif (PAM), a very short and structured sequence that is present in the viral genome but absent in the CRISPR spacer. Thus, the CRISPR-Cas system defends the host cell through a highly specific nucleotide interference machinery, in which the CRISPR ribonucleoprotein (crRNP) complexes silence or degrade foreign genetic material by cleaving RNA or DNA.

Two main classes of CRISPR-Cas systems have been identified, each including three types, according to the Cas proteins involved. Class 2 consists of 3 types, including the renowned CRISPR-Cas9 system, and is characterized by the presence of a single effector protein for interference (Cas9 for type II, Cas12 for type V, and Cas13 for type VI). Class 1 CRISPR-Cas systems, which includes types I and III, are characterized by multiprotein complexes with nuclease activity against either RNA or DNA [14].

In addition to their immune function, the CRISPR-Cas systems are “adaptive”: they can incorporate fragments of invasive sequences as new spacers. This adaptation is mediated by two specific Cas proteins shared by all CRISPR-Cas systems, the Cas1 and Cas2. This mechanism ensures bacterial adaptation to the effective viral populations they are exposed to [14, 15]. CRISPR-Cas systems have been thoroughly reviewed by other authors [1, 12, 13, 15]. This highly efficient and specific machinery for nucleotide sequence recognition and processing has been leveraged for diverse biotechnological tools as will be discussed later.

## The use of internal CRISPR diversity for *M. tuberculosis* complex genotyping

**Fig. 1:**
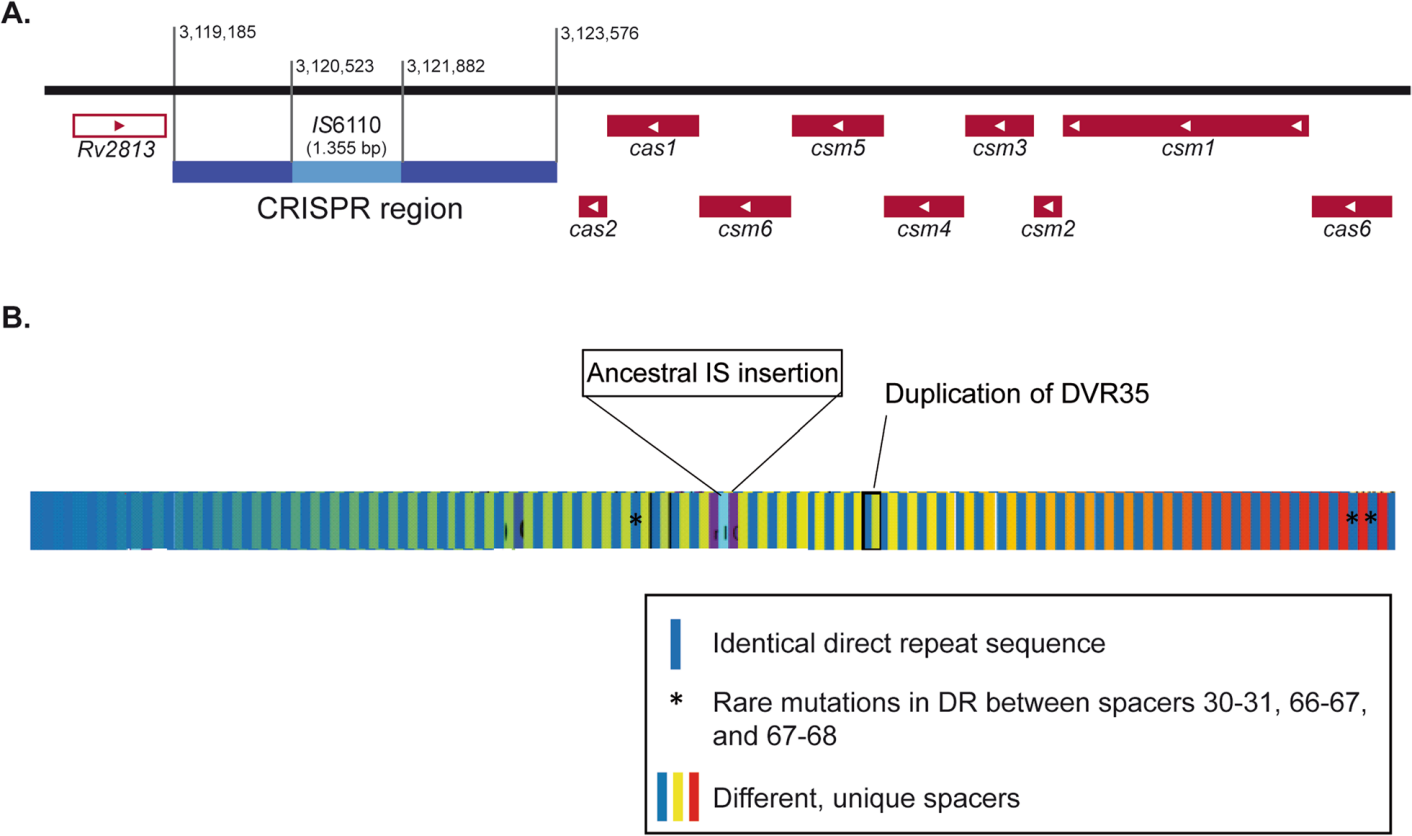
CRISPR locus in *Mycobacterium tuberculosis.* **A** Schematic representation of the genomic region containing the CRISPR-Cas genes in the reference strain H37Rv. The CRISPR region comprises 4392 bp including the *IS6110* copy inserted. Cas proteins are encoded upstream regarding the transcription direction on the reverse strand. **B** Representation of the reconstructed ancestral CRISPR locus. Direct repeats (DR), represented in dark blue, are interspersed with the spacer sequences, each having a unique nucleotide sequence. Although the DR sequence is highly conserved, three of them harbor rare mutations indicated by asterisks. Around the *IS6110* insertion, the DR are truncated, still allowing the fixation of standard spoligotyping probes pointing to the adjacent spacer. Of note, the duplication of DVR35 is not in tandem and lies next to DVR41

A CRISPR locus, also called direct repeat (DR) region or “internal CRISPR”, is present in almost all MTBC strains (Fig. 1A). As previously mentioned, CRISPR locus was identified in *M. tuberculosis* as early as 1993 [5] and is characterized by the repetition of identical DRs interspaced by unique sequences called spacers or variants [2]. The combination of one direct repeat and one variant is referred to as a Direct Variant Repeat (DVR). Based on the highly conserved nature of the spacer sequences, the CRISPR locus was immediately proposed as the target of a first-line molecular epidemiology tool termed spoligotyping [6, 16]. The technique involves the amplification of the spacers using primers targeting the extremes of the DR region, and hybridization of the product in a membrane with immobilized complementary probes. 43 spacers were selected for the assay, to discriminate between *M. bovis* and the most frequently isolated *M. tuberculosis* strains. Spoligotypes mainly evolve through the loss of these spacers and provide a binary barcode of 43 digits for each isolate, indicating the presence or absence of the corresponding spacer. This technique was able to discriminate samples from different locations, constituting an interesting first-line approach to explore the likely transmission events. It also helped identify relatedness between samples, leading to the definition of spoligotype families (such as East-African-Indian, African, Beijing, etc.) due to its relatively slow evolutionary rate. These families are highly congruent with single nucleotide polymorphism (SNP)-based lineages [17]. Altogether, spoligotyping allowed the first description of worldwide MTBC diversity [18] and still contributes to the characterization of *M. tuberculosis* [19].

Recent exploration of CRISPR diversity in the species belonging to the MTBC using next generation sequencing data showed that the ancestor of *M. tuberculosis* carried 68 different spacers, including one DVR that was duplicated [20] (Fig. 1B). Another interesting characteristic of this ancestral version of MTBC is the presence of an insertion sequence, the *IS6110*, which is conserved in most of the present isolates (Fig. 1). These analyses confirmed the overall conserved structure and sequence of the CRISPR locus in MTBC and its unique evolutive path mediated by mobile IS elements, deletion of DVR blocks and introduction of SNPs, rather than by classical CRISPR adaptation. In addition to the classical presence/absence of spacers analyzed by spoligotype, these changes detected by whole genome sequencing (WGS) and phylogenetic reconstruction, also followed an evolutionary pathway that overlaps with the current MTBC lineages. For instance, modern Beijing family strains belonging to lineage 2 harbor a large deletion in most of the spacers and part of the Cas genes related to an *IS6110* transposition event [20]. This study also showed that, except Lineage 2 strains, most clinical isolates retain a full set of Cas proteins, as already identified for several reconstructed genomes [21].

As a comprehensive and easily comparable method for global MTBC genotyping with a very restricted size of the genome explored, spoligotyping has been recently incorporated in the targeted deep-next generation sequencing technique named *deeplex*, providing high sensitivity and specificity of molecular diversity investigation [22]. In addition, several bioinformatic tools for the in silico retrieval of spoligotype from next-generation sequencing data have been developed [23, 24].

## Hypothetical impact of CRISPR in *M. tuberculosis* immunity to foreign DNA

Because *M. tuberculosis* has a relatively conserved CRISPR locus and diversity was found to be unrelated to virulence, it was long assumed to have no impact on bacterial physiology. CRISPR-Cas was thought of as a completely neutral locus, a distant ‘‘memory of past genetic aggressions” as described by Vergnaud in their pioneering work [12]. However, it has been recently described that MTBC strains carrying the complete set of Cas proteins can degrade artificial plasmid DNA sequences homologous to CRISPR spacer sequences, questioning this view [25].

Almost all MTBC strains present a single type IIIa CRISPR-Cas system [21] (Fig. 1A). A remarkable exception is *M. canettii*, an environmental MTBC member that only occasionally infects humans in the Horn of Africa, which can harbor diverse class 1 CRISPR-Cas types depending on the strain [26]. Because CRISPR-Cas systems were not found in non-tuberculous mycobacteria, these regions were probably acquired by horizontal gene transfer from other environmental bacteria in the ancestor of MTBC sensu stricto and *M. canettii*.

This type IIIa CRISPR-Cas system found in MTBC sensu stricto has long been thought to be inactive, both regarding adaptive and immune functions. The hypothesis that this locus is not essential is partly supported by the existence of strains with fully deleted CRISPR [27]. Yet the prevalence of these strains is very low. A way to evaluate the functionality of a locus is to measure its expression. Transcriptomics have been undertaken in a variety of culture conditions including the use of glycerol or glucose as nutrient sources, in exponential or stationary phases, in standard conditions, acidic conditions or in rifampicin (RIF)-induced latency [28–30]. In a retrospective analysis of the transcriptomic datasets generated under these various conditions, CRISPR and Cas genes, except Cas1 and Cas2, were always at least slightly expressed, and this expression was stronger under latency/dormancy states (G. Refrégier, unpublished results). These observations are in line with other reports [25, 31].

Regarding adaptive function, Cas1 and Cas2 not only presented poor “sense transcript” levels, but they harbored a level of “antisense transcripts”, suggesting that no proteins can be produced. Cas1 and Cas2 have not been detected in *M. tuberculosis* proteomes [32, 33], even in under hypoxic stress [34]. These observations suggested that the adaptive function of CRISPR in MTBC is inactive which is in line with the lack of newly acquired CRISPR spacers. So far, there is no evidence indicating that *M. tuberculosis* acquires phage resistance through the CRISPR system, even in experimental conditions [35, 36], and spacer sequences do not match any known mycobacteriophage sequences [21, 31].

In contrast, regarding the immune function, the transcription of Cas genes other than Cas1 and Cas2 and their detection in proteomic studies [33] suggested that the defense mechanism could be at least partially active. Accordingly, a recent study tested the permanence of a plasmid after transformation, carrying spacer sequences that are present in the H37Rv strain. The authors showed that only plasmids devoid of any spacer sequence were efficiently transfected and that the more spacer sequences of the *M. tuberculosis* CRISPR, the less this plasmid could be retrieved from transformed bacteria [25]. These authors also described that processed crRNAs were detected [25, 31], which likely contribute to the formation of the multiprotein crRNP complex in a Cas6 dependent fashion [25]. Transcription of CRISPR-Cas site seems to be regulated, and it has been shown that the MTBC CRISPR locus can be induced by cyclic di-adenylate [31]. Grüschow et al. recently reconstructed the Type III CRISPR-Cas system of *M. tuberculosis* in *E. coli* and found that specific immunity against mobile genetic elements could be mediated by cyclic oligoadenylate signaling produced by the interference complex. Immunity is mediated by the ancillary RNAse activity of Csm6, rather than by the nuclease activity of the CRISPR-Cas complex [37].

Non-canonical roles of mycobacterial Cas proteins have also been shown through the heterologous expression of *M. tuberculosis* Cas1 in *M. smegmatis*, which led to enhanced susceptibility to anti-tubercular drugs and DNA damaging agents [36]. Other authors have shown that Csm1, 2, 3 and 5 along with Cas6 are secreted by *M. tuberculosis* [38] and that these proteins have immunomodulatory properties [38, 39].

Collectively, these lines of evidence indicate that, in contrast to previous assumptions, MTBC CRISPR-Cas system retains at least some functionality: crRNA processing, crRNP assembly, target nucleotide recognition and target degradation by ancillary RNAse activity are detected under certain conditions in *M. tuberculosis*. Some gene silencing strategies take advantage of the endogenous activity of Cas proteins in *M. tuberculosis* as will be discussed later [40, 41].

## Applications of CRISPR-Cas technology for tuberculosis diagnosis and MDR-tuberculosis detection

Basic knowledge about the CRISPR-Cas system in prokaryotes led to an impressive development of diverse technological applications in recent years, and CRISPR-Cas-based molecular diagnostics of infectious diseases is a rapidly expanding field. Despite the molecular biology toolkit for tuberculosis diagnosis significantly improved in the last decade, several challenges remain. The nucleic acid amplification tests (NAAT) that are currently available have a good performance, with sensitivity and specificity surpassing 90% and 95% respectively for respiratory samples from people suspected of tuberculosis, outperforming the traditional microbiological methods. However, their sensitivity remains low in paucibacillary patients such as children [42] and HIV co-infected people [43]. The next-generation GeneXpert Ultra test has a sensitivity of 73% and a specificity of 97% in sputum samples of children suspected of pulmonary tuberculosis compared to the microbiological reference standard, and an 88% sensitivity and a 95% specificity in sputum samples of adult patients living with HIV. Sensitivity in non-respiratory samples such as cerebrospinal and pleural fluids is between 50 and 70% [44]. The performances of these tests are significantly lower if clinically diagnosed cases are included in the comparison, indicating that an important number of patients do not have microbiological confirmation of their tuberculosis, especially in the above-mentioned vulnerable groups. For this reason, the World Health Organization reinforces the need of more accurate, fast, high throughput and accessible methods for tuberculosis diagnosis at the point of care (POC) and for the detection of resistance conferring mutations [45].

**Fig. 2:**
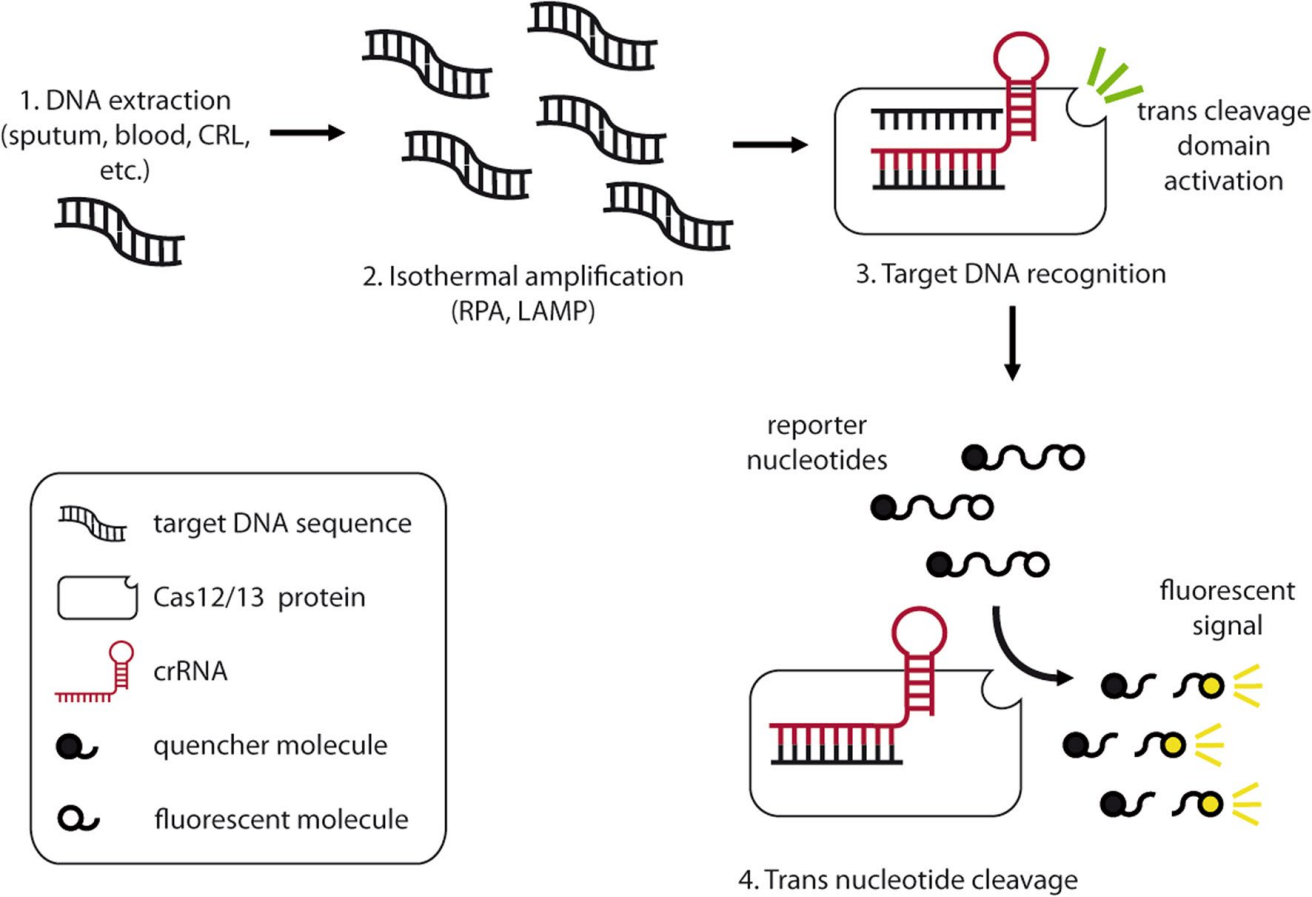
CRISPR-Cas based methods for the diagnosis of tuberculosis. General workflow for the detection of target DNA sequences is represented. After DNA extraction (1), sample is subjected to a nucleotide amplification step through an isothermal method, which has lower requirements of equipment compared to traditional PCR (2). Target DNA is recognized by the crRNA coding the complementary sequence which forms a crRNP with Cas12 or Cas13 molecules, leading to the activation of their trans cleavage domain (3). Synthetic reporter oligonucleotides are released from the quencher molecule after cleavage in trans by the crRNP and the fluorescent signal is read (4). *RPA* recombinase polymerase assay, *LAMP* loop-mediated isothermal amplification

Because oligonucleotides can be easily engineered, Cas-based technologies are a powerful tool for the detection of specific nucleotide sequences. In addition, the discovery of the so-called trans-cleavage activity against by-standing nucleotides in some of the Cas proteins boosted the development of several highly specific detection and signal amplification methods for molecular diagnosis (Fig. 2) [46]. Several strategies using type V and type VI Cas proteins have been developed, based on their ability to collaterally cleave non-targeted nucleic acids upon activation by the recognition of the specific nucleotide sequences complementary to the crRNA. Cas12 has trans-cleavage activity against single stranded (ss)DNA and Cas13 against ssRNA, which is exploited to cleave engineered nucleic acids that fluoresce after the release of a physically attached quencher molecule. Cas-mediated nucleotide recognition and signal amplification were combined with PCR or isothermal NAAT to develop several tests for the diagnosis of infectious diseases. This method applied in a SARS-CoV2 detection kit received an emergency approval by the American Food and Drug Administration [47]. CRISPR-Cas based tests not only showed femtomolar (10^−15^ M) sensitivity and high specificity, far below the limit of detection of traditional PCR-based methods. They showed good performance in terms of turnaround time and ease of use. Moreover, isothermal amplification methods such as recombinase polymerase assay (RPA) or loop-mediated isothermal amplification (LAMP), which can be combined with CRISPR detection in lateral flow immune-chromatography, make these approaches attractive as POC tests that do not need sophisticated equipment for readout [46]. One-pot detection has already proved possible using LAMP and a thermostable Cas12b with very high specificity [48].

Based on similar designs, several experimental tests were evaluated for the diagnosis of tuberculosis, which were recently reviewed elsewhere [49]. These methods target conserved genes from the *M. tuberculosis* genome such as the *IS6110* and combine isothermal pre-amplification with Cas12 or Cas13-based detection methods. Their advantage relies on the ease of implementation, especially on biological samples such as sputa, and turn-around time. The specificity of these techniques has indeed been evaluated against non-tuberculous mycobacteria [50, 51] and in complex biological samples [50, 52].

Interestingly, one of these tests was able to detect *M. tuberculosis* DNA in clinically diagnosed tuberculosis patients which had no microbiological confirmation by the methods endorsed by WHO [52] indicating that the detection threshold in extra-pulmonary and paucibacillary tuberculosis cases can be significantly lowered. Moreover, in this retrospective study applying the newly developed CRISPR-TB test for the analysis of circulating cell-free (cf) DNA in a cohort of immunosuppressed children (median [IQR] age: 2 [0.8–5.1]) that did not receive anti-tuberculous treatment, Huang et al. found that the risk of mortality was higher among those who had a positive CRISPR-TB signal. This suggests that early detection of *M. tuberculosis* through blood cfDNA testing could be critical to reduce mortality rates [52]. Future prospective cohort studies will certainly shed light on the clinical relevance of the low abundance *M. tuberculosis*-cfDNA. Considering that respiratory samples in children are difficult to obtain, this blood-based method has an additional advantage. In addition, unlike what is observed with NAATs based on respiratory specimens, cfDNA levels declined after anti-tuberculous treatment, endorsing its potential use for treatment monitoring.

Massive application of CRISPR technology for diagnosis must overcome several challenges in the future which are summarized in Box 1. First, the tests developed so far rely on DNA extraction methods which require laboratory equipment that is not available in resource-constrained settings or at the POC. Next, molecular diagnosis methods for tuberculosis have the additional challenge of high cross-contamination rates and biosafety requirements for culture-based methods. These limitations were solved in part in closed automated systems such as the GeneXpert but involving relatively high costs for developing countries. With very promising results, Huang et al. adapted the CRISPR-TB method to a lateral immunochromatographic POC test which can improve the microbiological diagnosis. Regarding affordability, this technology combined with isothermal amplification methods can lead to a significant reduction in equipment costs. However, non-PCR pre-amplification step would require a higher cost in recombinant enzymes and more complex primers compared to traditional PCR [46].

Tuberculosis has witnessed an increase in MDR (multidrug resistance) cases, which often requires prolonged treatment with expensive and toxic drugs, thus posing higher risks of treatment failure. Drug-resistance acquisition in *M. tuberculosis* relies exclusively on SNPs and insertion-deletion mutations (indels). Thus, an interesting application of CRISPR technology would be the detection of drug-resistant *M. tuberculosis* clones. There are, however, two major hurdles to overcome. In the first place, unlike for PCR-primers, currently there are no automated bioinformatic methods for the designing of crRNAs for SNP-level discrimination. The second limitation is related to the difficulty of designing multiplexed tests with CRISPR-Cas systems due to the unspecific nature of trans cleavage activity used for readout. This is a critical constraint, because sensitivity for drug resistance conferring mutations depends on the number of target mutations to be tested [53]. For this reason, current PCR-based kits target 4–5 mutations for the detection of rifampicin only. Multiplexing strategies are currently in development, but the possible solutions imply higher cost or assay complexity that undermine some of the advantages of CRISPR-based testing [46].

Future developments should point to complement or improve the currently available methods. WHO endorsed WGS for the diagnosis of tuberculosis and DR-tuberculosis, although this technology applied to biological samples is not as sensitive as amplification-based methods. Metagenomic [54] and targeted amplification plus next generation sequencing approaches might have a role in the near future [55]. In this line, a CRISPR-based amplification and sample preparation for indexing that can be applied in targeted sequencing with next generation sequencing has been recently described [56]. The forthcoming developments along with those described herein are promising for improving tuberculosis diagnosis.

Box 1. Challenges for the massive application of CRISPR in tuberculosis diagnosis

1. DNA extraction methods.
2. Automation and closed system to prevent crossed contamination.
3. Development of POC tests such as lateral immunochromatography.
4. Affordability.
5. Automated crRNA design tools for SNP-level discrimination.
6. Multiplexing.

## The use of CRISPR for targeted gene modification in mycobacteria

Traditionally, genome editing in bacteria has included a wide range of laborious and multi-step methods. The discovery of the CRISPR-Cas-mediated technologies facilitated genome editing in these microorganisms due to its simplicity and programmability. This is particularly relevant for functional genomic analysis because in the slow-growing *M. tuberculosis* classical methods for genome editing or silencing are particularly inefficient and tedious. Several CRISPR-Cas-based strategies for genetic modification have been developed, and some of them take advantage of endogenous molecules of the target organisms. Unlike most bacteria, mycobacteria have DNA repair systems including the error-prone Non-Homologous End.

Joining (NHEJ) mechanism [57], and the Homologous Directed Recombination (HDR), which uses a homologous donor DNA template to repair the DNA damage [58]. These endogenous systems allow the use of Cas-based genetic modifications in *M. tuberculosis* because they quickly repair Cas-induced double strand breaks which can lead to bacterial death.

For these reasons, CRISPR/Cas systems have evolved as one of the main genome-editing tools in mycobacteria, expanding the horizons for the exploration of their biology.

In general, these strategies rely on the transfection of one or more plasmids encoding the engineered Cas proteins and the guide RNA (gRNA) for the target sequence, coupled with accessory enzymes and oligonucleotides depending on the technique. Here, we summarize the main CRISPR-Cas-mediated techniques used in genome editing and expression modulation in mycobacteria (Table 1).

**Table 1:** Techniques for CRISPR/Cas-mediated genome editing in mycobacteria

Technique	Cas (Class, type)	Keys features	Application	Mycobacteria	References
CRISPR interference (CRISPRi)	dCas9 (class 2, type III)	– Mutant Cas9 from *Staphylococcus* without endonuclease activity	Regulation of gene expression	*M. smegmatis* *M. tuberculosis*	Choudhary et al. [60] Rock [62]
		– Interferes with target gene transcription			
CRISPR-assisted-recombineering	Cas12a (class2, type Va)	– Co-transfection with ds or ssDNA with the desired mutation	Generation of point mutations and indels	*M. smegmatis*	Yan et al. [65]
		– Coupled with Mycobacteriophage Che9c to increase the efficiency of recombination			
		– Induces a DSB distal to PAM (staggered ends)			
CRISPR-FnCpf1-assisted-NHEJ	Cas12a (class2, type Va)	– Coupled with overexpression of NHEJ proteins and inhibition of RecA-dependent HR-mediated repair	Generation of deletions and double mutations	*M. marinum* *M. smegmatis* *M. tuberculosis*	Sun et al. [66] Yan et al. [67]
		– Target sequence coded in the FnCpf1 plasmid			
		– Induces a DSB distal to PAM (staggered ends)			
		– Cleaves first the non-target strand			
CRISPR1-Cas9	Sth1dCas9 (class 2, type II)	– dCas9 with nuclease activity restored	Generation of indels	*M. marinum* *M. smegmatis* *M. tuberculosis*	Meijers et al. [68]
		– Induces a DSB proximal to PAM (blunt ends)			
Type III CRISPR system	Csm/Cmr (class 2, type III)	– Endogenous CRISPR-Cas system replaces target genes by means of a gRNA and a HDR template	Gene knock in/KO Single and multiple gene RNAi	*M. tuberculosis*	Rahman et al. [41]
		– Cleaves RNA and ssDNA			
Base editing system	nCas9 (BE) (class 2, type V)	– Modified Cas9 fused to APOBEC1 and UGI for the conversion of single bases without altering the surrounding sequence (lower off targets)	Site-directed mutagenesis	*M. tuberculosis*	Ding et al. [72]
		– Efficient G:C to A:T conversion in target genes			
CRISPR-guided DNA polymerase system (CAMPER)	nCas9 (class 2, type V)	– Coupled with an error prome DNA polymerase A	Site-directed mutagenesis	*M. smegmatis* *M. tuberculosis*	Feng et al. [73]
		– Lower off-targets than Cas9			
		– Induces single strand breaks guided by the gRNA, followed by nick translation and induction of random substitutions			

*DSB* double strand breaks, *PAM* protospacer adjacent motif, *NHEJ* non-homologous end joining, *HR* homologous recombination, *gRNA* guide RNA, *HDR* homology directed repair, *ssDNA* single stranded DNA, *APOBEC* cytidine deaminase APOBEC1, *UGI* uracil DNA glycosylase inhibitor

The CRISPRi approach was developed for targeted gene regulation in microorganisms. It relies on the expression of an endonuclease deficient Cas9 (dCas9). The dCas9 has the ability to interfere with target DNA transcription through a small guide RNA (sgRNA) encoding the complementary sequence, leading to the repression of the target gene [59]. This approach has been used in mycobacteria for knocking-down the expression of certain genes in *M. smegmatis* and *M. tuberculosis* [60, 61]. A codon-optimized dCas9 of *Streptococcus pyogenes* was used in this system, which is stable for up to two weeks without any toxic or off-target effects in mycobacteria. CRISPRi holds the promise of enabling anti-tuberculosis antibiotic discovery, expanding the range of biologically attractive targets [62]. In addition, CRISPRi has also been used for high throughput screening of genes affecting fitness when partially inhibited [63].

The CRISPR-assisted-recombineering approach relies on the homologous recombination (HR) of a DNA template with a target DNA by programmable nucleases from the CRISPR-Cas systems. Recombineering was successfully used for genetic manipulation in mycobacteria. It combines the mycobacteriophage Che9c-based recombineering method [64] coupled with the CRISPR-Cas12a, an endonuclease (type V-A) of the class 2 CRISPR-Cas system. CRISPR-Cas12a-assisted recombineering is used to generate point mutations, deletions, and insertions in *M. smegmatis* [65].

The CRISPR-FnCpf1-assisted-NHEJ relies on the use of the CRISPR-Cas12a (also known as FnCpf1) that recognizes the target gene using a guide RNA and generates DNA double-stranded breaks (DSBs) which are in turn repaired by the non-homologous end joining (NHEJ) system. NHEJ tends to produce indels at the junctional site and the frameshift mutation disrupts the targeted gene. This approach was used to generate deletions in different mycobacteria [66, 67]. It is also capable of producing large-scale random double mutations in *M. tuberculosis* via the inhibition of RecA-dependent HR-mediated repair in addition to overexpression of the mycobacterial NHEJ proteins [67].

The CRISPR1-Cas9 relies on restoring the enzymatic activity of the catalytically inactive *Streptococcus thermophilus* CRISPR1-Cas9 (Sth1dCas9). This system consists of a single plasmid that contains the Sth1Cas9 along with the guide RNA without the need to introduce NHEJ proteins. This approach form highly efficient and precise DNA breaks and indels without any off-target effects [68].

The endogenous type III-A CRISPR system of *M. tuberculosis* was leveraged for gene knock-in/knockout (KO) and for single/multiple gene RNAi to precisely dissect the functions of specific genes. This approach relies on the use of 40-bp gRNAs targeting the coding strands coupled with an HDR template to insert or replace specific genes of *M. tuberculosis* and also for massive mutagenesis and screening of genes related to bacterial growth [41].

The base editing system provides a novel strategy for precise genetic manipulation without the need to introduce DSBs or donor DNA templates [69, 70]. This system uses a plasmid which expresses RecX and NucSE107A to repress HR and mismatch repair DNA repair pathway combined with a second plasmid which encodes a codon-optimized fusion protein named BE, consisting of a modified Cas9 fused to the cytidine deaminase APOBEC and a uracil glycosylase inhibitor (UGI). BE deaminates the cytidines to uracils on the target within a small window, which in turn are converted from G:U to A:T by a mismatch repair system at desired sites in the genome [71], enabling efficient G:C to A:T base pair conversion at desired sites in the *M. tuberculosis* genome [72].

One state-of-the-art approach for functional genomic analysis is the use of the CRISPR-guided DNA polymerase system (also known as CAMPER) [73]. It relies on a sgRNA-guided xCas9 nickase (Cas9 variant recognizing NGN as the PAM sequence) along with an error-prone DNA polymerase. This system was able to introduce random substitution mutations within a 80 bp long editing window enabling the detection of novel drug-resistant mutants [73].

Many of these approaches are scalable and combined with high throughput screening of the resulting phenotype based on artificial intelligence algorithms will certainly shed light into the metabolic networks underlying drug-resistance, virulence, and immune modulation by *M. tuberculosis* in the next years.

## CRISPR in the study of the immune response against *M. tuberculosis*

CRISPR-based technologies are also expected to shed light on the intricate host–pathogen interaction in tuberculosis. Macrophages play a critical role in the immune response to *M. tuberculosis* through granuloma development and mycobacterial infection containment. Studies of macrophage function have been hampered due to the low efficiency of traditional genetic manipulation methods and the intrinsic limitations of widely used macrophage-like cell lines [74]. Gene KO can be obtained by delivery of CRISPR-Cas9 RNPs in primary human and murine myeloid cells [75] as well as in induced Pluripotent Stem Cell (iPSC)-derived macrophages [76]. A conditionally immortalized macrophage system based on the ectopic expression of the transcription factor ER-Hoxb8 in hematopoietic progenitors from Cas9-expressing transgenic mice has proven to be useful in dissecting the impact of specific host genes during *M. tuberculosis* infection [74]. High-throughput, pooled-based CRISPR-Cas screening approach to identify essential genes required for macrophage function and viability has recently been developed. These approaches led to the discovery of previously unidentified regulators of the pivotal transcription factor NFκB [77], manipulation of metabolic pathways by the pathogen [78], and mediators balancing macrophage survival with controlled intracellular *M. tuberculosis* vs. macrophage death and uncontrolled intracellular growth [79]. In addition, Lai et al. simultaneously screened CRISPR-KO and CRISPRi-knock down libraries allowing to identify essential pathways in macrophages that are missed by KO screening [78, 79]. With an interesting approach, a recent report shows that *M. tuberculosis* genes targeted by CRISPRi can be specifically repressed in intracellular bacteria infecting THP-1 human macrophage-like cells [80].

Study of lymphocytes through CRISPR-KO and CRISPRi has been centered in T lymphocyte transformation [81]. Because sustained expression of Cas9 can lead to off-target effects and potential oncogenesis, transient transformations are the preferred approach. Technical improvements are still needed to tackle current limitations such as transformation efficiency and stability, resistance against the most frequently used lentiviral vector or difficulty to ensure diploid transformation, but new developments are on its way [81, 82]. In addition to loss-of function screens, CRISPRa has been developed for enhancing the expression of target genes through a Cas9 fused to transcriptional activators and sgRNAs encoding promoter of transcription start regions [82]. CRISPRa and CRISPRi screenings can be matched for a comprehensive analysis of immune cell functionality [83]. As far as we know, lymphocyte biology in tuberculosis has not been interrogated through CRISPR-based tools, but these approaches are expected to shed light into the protective and pathogenic responses, the precise role of type I and type II IFNs as well as pathways involved in lasting immunological memory against mycobacteria. In the context of tuberculosis, it would be interesting to analyze the drivers of peripheral blood lymphocyte hypo-responsiveness in patients with active disease and the possibility to revert deleterious effects in patients with Mendelian susceptibility to mycobacterial diseases, which could be useful for the rational design of novel therapies.

Polymorphonuclear neutrophils are thought to have a role in granuloma formation as well as in host tissue damage in tuberculosis. These cells are short-lived and hyperreactive, making them difficult to manipulate ex vivo. Interesting findings were obtained using Cas9 generated KO zebrafish. It was shown that protection against *Mycobacterium marinum* infection is mediated by the inflammasome-IL-1β axis [84, 85], and that the micro RNA miR-206 inhibits neutrophil recruitment and retention via *cxcl12a* and *cxcr4* genes [86]. In vivo conditional KO and knock downs could also be useful to dissect the mechanisms involved in neutrophil-mediated immunity against mycobacteria.

Host-directed therapies (HDTs), aiming to boost an adequate host immune response to mycobacterial infection constitutes another avenue of treatment. Using high-throughput CRISPR-KO and CRISPRi screenings, it is possible to identify perturbations that improve the survival of human phagocytic cells infected with intracellular pathogens [78, 79]. Identification of genes and biological pathways associated with enhanced human macrophage survival and limited intracellular growth of *M. tuberculosis* can lead to potential HDT targets. The final goal of these approaches is to use small-molecule inhibitors to modulate detrimental signaling pathways like type I interferon, which is associated with active tuberculosis [87], or newly linked metabolic pathways which could improve the immunological outcome. Human peripheral blood lymphocytes have been successfully manipulated for adoptive transfer therapy in patients with refractory cancer [88]. Through a Cas9-RNP nucleofection method, a recent report shows that human primary monocytes can be knocked out ex vivo for a specific target, retaining their ability to differentiate into functional monocyte-derived macrophages and dendritic cells [89].

CRISPR-Cas9 has also been used to provide a novel make up for a century old vaccine, the BCG. BCG is used in high burden countries to prevent disseminated forms of tuberculosis in children, but it is not protective against pulmonary tuberculosis [90]. A recombinant BCG expressing a genetically detoxified subunit A of heat-labile toxin from *Escherichia coli*, proved to induce improved protection against *M. tuberculosis* in mice, including a hypervirulent Beijing strain [91]. CRISPR/Cas9 has also been used to remove the antibiotic resistance marker for recombinant construction, so the unmarked auxotrophic BCG strain could be used as a vaccine in humans [92].

Collectively, it is expected that CRISPR-based functional genomics, combined with high resolution methods for output screening such as single cell RNA-sequencing will provide a clearer view of the complex interaction networks in tuberculosis immunity. A better understanding of host and pathogen genes involved in the protective and pathogenic immune responses is urgently needed for a better management of tuberculosis, and researchers have now the possibility to study them in detail, manipulating both sides with high accuracy.

## Conclusions

Tuberculosis is an ancient disease that still afflicts global health and novel approaches for the prevention, diagnosis, treatment and monitoring are urgently needed. Fundamental research led to a better understanding of the remnant activity of *M. tuberculosis* CRISPR locus, that can be leveraged for the development of innovative technological applications.

The CRISPR-Cas based technologies boost our possibilities to further explore the biology of *M. tuberculosis* and its interaction with host immune system, which would, in turn, allow the discovery of novel anti-tuberculosis drugs, HDTs and vaccines. New diagnostic methods could tackle underdiagnosis. Altogether, the CRISPR-revolution will revitalize the fight against tuberculosis with more research and technological developments.

## Data Availability

Not applicable.

## Abbreviations

CRISPR: Clustered Regularly Interspaced Short Palindromic repeats
Cas proteins: CRISPR-associated proteins
cfDNA: Cell-free DNA
CRISPRi: CRISPR interference
crRNA: CRISPR RNA
crRNP: CRISPR ribonucleoproteins
DR: Direct repeat
DSB: Double strand break
DVR: Direct variant repeat
gRNA: Guide RNA
HR: Homologous recombination
HDR: Homologous directed recombination
HDT: Host-directed therapies
indel: Insertion-deletion mutations
INH: Isoniazid
iPSC: Induced pluripotent stem cell
*IS6110*: Insertion sequence 6110
KO: Gene knock out
LAMP: Loop-mediated isothermal amplification
MTBC: *Mycobacterium tuberculosis* Complex
NAAT: Nucleic acid amplification tests
NHEJ: Non-homologous end joining
PAM: Protospacer adjacent motif
POC: Point of care
RIF: Rifampicin
RPA: Recombinase polymerase assay
SNP: Single nucleotide polymorphism
UGB: Uracil glycosylase inhibitor
WGS: Whole genome sequencing
ssDNA: Single stranded DNA
